# A 3D Printer in the Lab: Not Only a Toy

**DOI:** 10.1002/advs.202202610

**Published:** 2022-07-13

**Authors:** Vittorio Saggiomo

**Affiliations:** ^1^ Department of BioNanoTechnology Wageningen University Bornse Weilanden 9 Wageningen 6708WG The Netherlands

**Keywords:** 3D printing, automation, materials, modeling

## Abstract

Although 3D printers are becoming more common in households, they are still under‐represented in many laboratories worldwide and regarded as toys rather than as laboratory equipment. This short review wants to change this conservative point of view. This mini‐review focuses on fused deposition modeling printers and what happens after acquiring your first 3D printer. In short, these printers melt plastic filament and deposit it layer by layer to create the final object. They are getting cheaper and easier to use, and nowadays it is not difficult to find good 3D printers for less than €500. At such a price, a 3D printer is one, if not the most, versatile piece of equipment you can have in a laboratory.

## Graphical abstract

A short review on the multiple applications of consumer 3D printers in a laboratory setting. From simple downloading and printing to 3D design to making specialized 3D printing material to reprogramming the 3D printer as a robot to automate laboratory procedures.

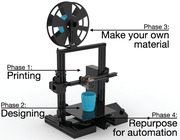

## 1 Introduction

“Why should we buy a 3D printer?” This is, unfortunately, the question too many students still get from their professors when asking to buy a 3D printer for the laboratory. And it is a very valid question. Although 3D printers are becoming more common in households, they are still under‐represented in many laboratories worldwide and regarded as toys rather than as laboratory equipment. This short review wants to change this point of view. If you are a student who got the “why?” question, share this mini‐review and discuss the possibilities opened by owning a 3D printer. If you are a PI, undecided about buying a 3D printer in the lab, read this mini‐review, or if you do not have time to read it: just buy one, you will thank me later.

This mini‐review focuses on Fused Deposition Modeling (FDM) printers and what happens after acquiring your first 3D printer. In short, these printers melt plastic filament and deposit it layer by layer to create the final object. They are getting cheaper and easier to use, and nowadays it is not difficult to find good 3D printers for less than €500. At such a price, a 3D printer is one, if not the most, versatile piece of equipment you can have in a laboratory, almost independently from a specific field. In the following paragraphs, you will see how a 3D printer can be useful in many different laboratories, from chemistry to biology, to material science, and more.

So, you bought your first 3D printer, assembled it, and now it is in the laboratory.^[^ ^1^ ^]^ What is next?

**Figure 1:**
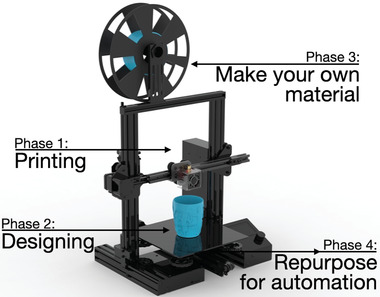
This mini‐review focuses on the four phases of owning an FDM 3D printer: printing, designing, materials, and automation.

There are four stages of owning a 3D printer as a scientific instrument: printing, designing, materials, and automation. In the following paragraphs, I will elaborate on each phase (**Figure** **1**).

## 2 Phase One: Printing

In the first weeks and with almost total certainty, the printer will be used to print a vast array of toys, figurines, and random objects. This is a normal learning process. During this time, users will learn how the 3D printer works and its lexicon, slice a 3D design, overhangs, bridging, brim, skirts, infill geometries, printing speed and temperatures, retraction settings, how to print different materials, and more. This jargon may be new to you, but do not despair, as 3D printers are for the consumer market, you will find plenty of valuable guides and videos on how to set up a 3D printer and print with it. This is an essential learning process; there will be plenty of failures, and each will be a valuable learning point.

**Figure 2:**
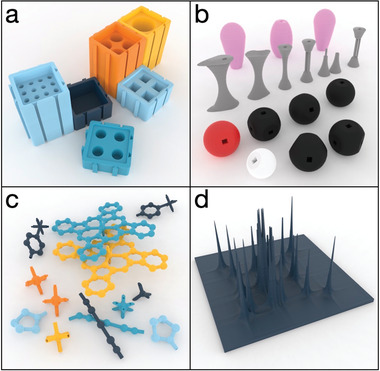
Examples of 3D printable designs: a) modular holders for various labware: NMR tubes, cuvettes, falcon tubes, downloadable from.^[^ ^21^ ^]^ b) Chemistry model sets which can be also downloaded and printed from.^[^ ^4^ ^]^ c) X‐ray structures,^[^ ^12^ ^]^ and d) NMR 2D spectra can also be converted to 3D printable models.^[^ ^13^ ^]^

Once the random printing is finished and users are acquainted with the 3D printing process, the second stage of this phase is to start printing valuable items for the lab (**Figure** **2**). And here, the possibilities are endless. Most probably, the first useful things 3D printed in any laboratory are holders for every single vial, tube, and cuvette you have in the lab.^[^ ^2^, ^3^ ^]^ There are plenty of designs, from NMR tube holders to magnetic‐ready Eppendorf holders for magnetic separation, freely available online from repositories like Thingiverse, Printables, or the NIH 3D printing repository. If you need a holder for something, it has probably already been designed by someone else, and it is ready to be downloaded, sliced for 3D printing, and printed (Figure 2a).

Even if you stop reading here, and your printer will only be used to print holders, you will already have the return on investment in a few months, considering how much sample holders cost.

But you will want more once you have dipped your toes in 3D printing. Scanning for literature at this point will return plenty of 3D printable objects: molecular structural models,^[^ ^4^, ^5^, ^6^ ^]^ corals,^[^ ^7^ ^]^ crystal structures, and 3D printed chips for obtaining crystals,^[^ ^8^ ^]^ which are valuable models for teaching, and give the student a physical model of chemical structures, and so on (Figure 2b). There are, for example, instructions on how to convert crystallographic data to 3D printable models (Figure 2c)^[^ ^9^, ^10^, ^11^, ^12^ ^]^ or even how to convert NMR spectra to 3D prints (Figure 2d).^[^ ^13^, ^14^ ^]^ 3D printing and NMR do not end here: you can print sample tubes for solid‐state NMR,^[^ ^15^ ^]^ whole NMR magnets,^[^ ^16^ ^]^ and even scaffolds for making NMR coils.^[^ ^17^, ^18^, ^19^ ^]^ A review on 3D printing in NMR can be found here.^[^ ^20^ ^]^

Fixing broken plastic laboratory objects has also become extremely easy, and many pipette holders or pipette parts, connectors, and desk organizers are freely downloadable from repositories.

The most heard criticism of this phase is that 3D printed materials are not stable to organic solvents compared to commercial laboratory plastic parts. Do not get me wrong; this is a valid criticism, and it is true. The most common 3D printed plastics, polylactic acid (PLA) and polyethylene terephthalate glycol (PETG), are dissolvable in chlorinated solvents. At the same time, acrylonitrile butadiene styrene (ABS) is soluble in acetone, which would make these materials not suitable for a laboratory environment. However, I argue that these materials are splash resistant. They will not magically disappear if one drop of solvent touches them. If you repeatedly flood your fume hood with organic solvents, I suggest revisiting the safety procedures of the laboratory for handling chemicals rather than buying chemical‐resistant holders. This boils down to rephrasing an ancient Sun Tzu quote: “know your 3D printer, know your material, and you will never be defeated.”

## 3 Phase Two: Designing

At a certain point, you will realize that the model you need is not available online, and your laboratory will enter the second phase of owning a 3D printer: Designing.

I cannot stress enough how significant it is for scientists to have at least a basic knowledge of 3D design, not only for 3D printing but also for making attractive figures for papers and presentations. Learning 3D design may look like a difficult task but, trust me, it is not that difficult, at least for simple designs. If you can sketch something on paper, you can design it yourself on a computer.

By designing your models, you unlock the full potential of 3D printing. Now, you can produce parts that are not commercially available, that are a perfect fit for your application or laboratory, or that are replacement or enhancement parts for other instruments there. This is the true power of rapid prototyping; you can design, print, test, and redesign an object in less than one day without ordering the part from a specialized online service—which, typically, requires weeks. For example, modifying an expensive centrifuge for separating large volume blood samples with a simple 3D printed add‐on.^[^ ^22^ ^]^ Creating something from scratch and having it in your hands a few hours later is an incredible experience.

Two scientific fields have greatly benefited from the advent of 3D printing and the possibility of rapid prototyping directly in the laboratory: microfluidics^[^ ^23^, ^24^ ^]^ and microscopy.^[^ ^25^ ^]^

Before the advent of 3D printing, making a microfluidic device was a cumbersome task: its steps involve making a master using soft lithography, molding and demolding polydimethylsiloxane (PDMS), alignment in case of multilayer microfluidics, and final plasma bonding of PDMS to glass, a process commonly known as replica molding. If the microfluidics did not work as predicted, the process would have been repeated multiple times to fine‐tune the microfluidic parameters. This process has changed with 3D printing, where various designs can be done and printed in a few hours, speeding up the research and lowering prototyping costs. One approach is to design and print the whole microfluidic device in 3D printable plastics.^[^ ^26^, ^27^, ^28^, ^29^ ^]^ Printing a single plastic block rather than having a mixed PDMS/glass device drastically increases its resistance to pressure.^[^ ^30^ ^]^ Moreover, the printing process can be halted during printing to add other objects inside the printed object to add new functionalities to the device,^[^ ^31^ ^]^ a process usually not possible in standard replica molding microfluidic fabrication. This process has been employed, for example, to add a PMMA observation window^[^ ^32^ ^]^ or even electrochemical detectors directly in 3D printed microfluidic devices.^[^ ^33^ ^]^

**Figure 3:**
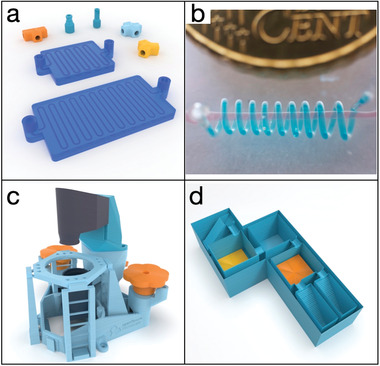
Example of 3D‐designed models. a) a set of microfluidics devices and connectors printable in various plastic materials.^[^ ^34^ ^]^ b) Method for manufacturing a PDMS microfluidic device using 3D printed dissolvable materials. Reproduced under the terms of the Creative Commons CC BY License.^[^ ^17^ ^]^ Copyright 2015, The Authors, published by Wiley‐VCH. c) Openflexure microscope, a fully 3D printable microscope.^[^ ^39^ ^]^ d) 3D printed cross‐section of labware for chemical reactions.^[^ ^48^ ^]^ A solution is moved into different cubes just by rotating the structure; in orange and yellow two different catalysts are directly 3D printed in the structure.

There is also an open‐source repository of 3D models for microfluidics, not only for devices themselves but also for all connections and adaptors (**Figure** **3**a).^[^ ^34^ ^]^

Another option for using a 3D printer to fabricate microfluidic devices is to use the solubility of some 3D printing plastics to our advantage. In 2015, our group printed ABS and embedded it into PDMS. When this PDMS/ABS block is placed in acetone, ABS dissolves, leaving an open channel inside the PDMS (Figure 3b).^[^ ^17^ ^]^ This method can make a single block PDMS microfluidic device, a standard and very well characterized material in the microfluidics field, using only a standard 3D printer and 3D printing material without the need for expensive or difficult to use instruments. This method has been applied to water‐soluble materials,^[^ ^35^ ^]^ or to manufacture interconnecting PDMS/glass microfluidic blocks.^[^ ^36^ ^]^ The FDM 3D printer can also be used to print a mold for PDMS replica molding fabrication.^[^ ^37^ ^]^

3D printing has also been used to fabricate paper microfluidics, using the 3D printing material for directing liquids on filter paper.^[^ ^38^ ^]^

The second field that has greatly benefitted from 3D printing is microscopy. The possibility of fast and cheaply prototyping microscope parts has democratized the field and opened the possibility of fabricating fully 3D printed microscopes. Probably the most common 3D printed microscope is the OpenFlexure (Figure 3c).^[^ ^39^, ^40^ ^]^ It uses a 3D printed stage to achieve stable movements of a few microns. Building on this kind of flexure, it is possible to 3D print a sub‐100 nm fiber alignment stage.^[^ ^41^ ^]^ This is impressive if you think that the stage is a single block of 3D‐printed plastic. Another recently developed 3D printable microscope is the PUMA.^[^ ^42^ ^]^

The use of 3D printers has also opened the field to the fabrication of Do‐It‐Yourself microscopes, where various 3D printed optical blocks can be put together for different microscopy set‐ups. The μCube^[^ ^43^ ^]^ is one of the first examples of this approach, and later the UC2 (You See Too) has improved the process, removing the need for tedious alignment of the blocks.^[^ ^44^ ^]^ Simply reshuffling the cubes on these set‐ups can transform a brightfield microscope into a fluorescent one in a few minutes.

Furthermore, 3D printing has also been used to fabricate a single‐molecule microscope: the miCube.^[^ ^45^ ^]^

An open microscopy database can be found here.^[^ ^46^ ^]^

And what if I told you that you can even design your chemical reactionware? This has been demonstrated multiple times by Cronin's group. The possibility of adding chemicals and external components such as filters or silica gel during the printing process allows the user to have whole synthetic and purification setups in a few hours. The versatility of 3D printing and the possibility of inserting external components in a 3D printed object allows for precise and reproducible synthetic and purification steps. Cronin's group proved this by printing cubes with different functionalities, and the single synthetic and purification steps are performed by simply turning the 3D printed object to move the solutions from one block to another (Figure 3d).^[^ ^47^, ^48^, ^49^ ^]^

The group has also shown that with polypropylene (PP) as 3D printed material, it is possible to make vessels stable to high temperatures and pressure.^[^ ^50^ ^]^

3D printing is also employed in analytical chemistry, another field that extensively uses 3D printing versatility for designing and printing separation devices, flow cells, mixers, concentrators, and more.^[^ ^51^, ^52^, ^53^, ^54^, ^55^ ^]^

A word of warning about materials if you plan to use them in contact with solutions of your interest. Commercially available 3D printing materials are rarely pure polymers. PLA, PETG, PP, etc., are not pure; they typically contain some copolymers, additives, and in the case of colored materials, pigments of some sort. Common 3D printing materials are formulated to be easily printable and for their mechanical properties, not for their purity. Even the same material from the same vendor can be chemically different from batch to batch. Do not assume that the materials are pure polymers, and if you plan to use them in contact with a solution of your interest, for example, for synthesis or analytical purposes, always test the material in advance. This can be done by NMR, or more quickly by placing the 3D printing material in the solvent you plan to use, letting it there for a few days, and then recording UV–vis spectra of the solution, looking for possible leakage of molecules. Both are fast methods that will give you at least an indication of the composition and the chances of something leaking from the material during the experiment.

Also for this phase, you will hear a classic criticism: “3D design is too difficult.” I am sure that you have encountered and survived calculus, thermodynamics, and quantum mechanics in your studies. Compared to these topics, 3D design is a walk in the park. Nowadays, there is plenty of easy‐to‐use 3D design software, the easiest one being Tinkercad, a free‐of‐charge web‐based software design. After a 10 min YouTube tutorial on how to use it, I can assure you that you will be able to make your first 3D designs in minutes.

For more complex designs, the most used softwares are Solidworks, Fusion 360, and FreeCAD. The first two are commercial, while the third one is free and open‐source software. They are more complex to use and require more training, but nothing impossible to learn. A more complex and powerful open‐source software for 3D design is the Blender.

Another option, if you like programming, is OpenSCAD, which uses a programming‐like structure and syntax to design 3D models.

Shapr3D is a recently developed software, initially designed for iPad+Stylus but now available for Mac and PC, free for academia and very intuitive to use.

And once more, 3D design is a helpful skill not only for 3D printing but also for making appealing scientific schemes and figures.

## 4 Phase Three: Make Your Own Material

You have mastered 3D printing and are getting used to 3D design. What is next? Having the possibility of making your own material. In FDM, this means dissolving the plastic in a proper solvent, adding the external material of interest, evaporating the solvent, shredding the plastic composite, and extruding it to form a printable filament using a commercial filament extruder like the FelFil Evo, Filabot, 3Devo, or by building your own filament extruder, like the Lyman Extruder V5.

This procedure is not easy, and the commercially available filament extruders are more expensive than a 3D printer because of the smaller market segment. However, you will have the possibility of making your own composite or multi‐material and printing in any shape you want, a thing impossible even to imagine only a decade ago.^[^ ^56^, ^57^, ^58^ ^]^

But why would you want to make your composite materials for 3D printing?

**Figure 4:**
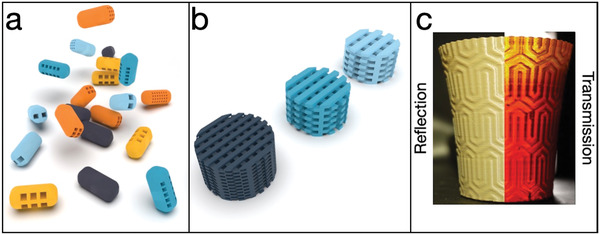
a) Examples of different materials and the impact of 3D printing in pharmaceuticals, where the structure of a pill can be changed at will for changing its dissolution time. b) A similar approach can be used for 3D printed catalysts, where the structure can be changed to have more or less surface area and catalysts present. c) A nanocomposite material that can mimic the Lycurgus cup, showing two different colors in reflection (green) and transmission (red). Reproduced under the terms of the Creative Commons CC BY License.^[^ ^110^ ^]^ Copyright 2020, The Authors, published by Beilstein.

One of the main fields that have taken advantage of this process is the pharmaceutical field, using specific formulations^[^ ^59^, ^60^, ^61^, ^62^ ^]^ and 3D printing personalized tablets with tailored drug release profiles (**Figure** **4**a).^[^ ^63^, ^64^, ^65^ ^]^ Studies have also been done to check the printability of composite pharmaceutical filaments.^[^ ^66^ ^]^

In medicine, composite 3D printable materials are being developed mostly for antimicrobial and medical‐grade materials for casts, prostheses, and objects that will be in contact with organs.^[^ ^67^, ^68^, ^69^, ^70^, ^71^, ^72^ ^]^

The possibility of inserting external chemicals in the 3D printing filament gives us the power to print augmented objects. This can be done for mechanical properties, for example, by using natural fillers, even hemp,^[^ ^73^ ^]^ to modify the printed object's mechanical properties.^[^ ^74^ ^]^ Metals,^[^ ^75^ ^]^ wood,^[^ ^76^ ^]^ and graphite^[^ ^77^ ^]^ have also been used to change the mechanical properties of 3D printed objects. But probably the most extreme case of this approach for mechanical properties is the embedding of a continuous fiber during the extrusion process.^[^ ^78^ ^]^ In this case, a continuous thread is inserted and driven inside the nozzle while it deposits the molten plastic so that the continuous thread is embedded in each deposited plastic line, greatly improving the mechanical strength of the final 3D printed part.

The 3D printed parts can be conductive by embedding conductive metals or carbon‐based nanomaterials in the filament.^[^ ^79^ ^]^ This is useful for example for making electromagnetic devices,^[^ ^80^ ^]^ (electrochemical) sensors,^[^ ^81^, ^82^, ^83^, ^84^ ^]^ circuits,^[^ ^85^, ^86^ ^]^ electrochemical cells,^[^ ^87^, ^88^ ^]^ and even batteries.^[^ ^89^ ^]^

The possibility of designing and printing different shapes is also useful for changing the aspect ratio of the 3D printed object, or to print objects with high surface area for analytical separation, membranes, and molecule capture. This approach, for example, has been shown fruitful for desalinization and (waste)water treatment,^[^ ^90^, ^91^, ^92^, ^93^ ^]^ and for gas capture using MOFs embedded in the 3D printing material.^[^ ^94^ ^]^

Another field that has greatly exploited 3D printing is catalysis (Figure 4b).^[^ ^95^, ^96^, ^97^, ^98^, ^99^, ^100^ ^]^ 3D printers, in this case, can be used not only to make (flow)reactors, mixers,^[^ ^101^, ^102^ ^]^ multi‐material parts,^[^ ^103^ ^]^ and so on, but also to embed catalysts directly in the 3D printed material.^[^ ^104^, ^105^, ^106^, ^107^ ^]^

FDM 3D printing has also been used in optics to make diffuse optics^[^ ^96^, ^97^ ^]^ and optic faceplates.^[^ ^108^ ^]^ Our group has also incorporated dichroic nanoparticles in a plastic filament for printing, for the first time, a dichroic object, in this case with an optical effect like the Lycurgus cup (Figure 4c).^[^ ^109^, ^110^ ^]^

This paragraph is only a short introduction to the multiple possibilities of making your own composite material for 3D printing, and it is not meant to be exhaustive, as there are other niches where composite 3D printing material will shine. However, I thought that it was necessary to show at least some opportunities arising from not only designing objects with specific features, for example, with high surface area, or for mixing capabilities, but also from coupling them with unique, application‐driven, composite materials made in the lab.

A word of warning here is about the FDM printing method. As the filament needs to be molten to be deposited on the 3D printing bed, it is heated, depending on the material, at more than 180 °C in the 3D printer hot‐end. This also means that every compound and material you have incorporated into the plastic will also be subjected to high temperatures, even if for a short amount of time. This high temperature may be detrimental, especially to organic molecules, and it should be considered when printing these composite materials. A way to monitor the degradation of organic molecules during the printing process is to dissolve the material after the 3D printing process and re‐characterize the organic compounds using NMR, MS, and UV–vis.

If you want to make a composite material and print it without being subjected to high temperature, you may want to investigate direct ink writing (DIW) 3D printing. This 3D printing method uses a syringe for depositing a paste in a layer‐by‐layer fashion, similar to FDM. Stereolithography (SLA) 3D printers, sometimes called “resin” printers, are also seen as non‐high temperature printers. However, this is misleading as the methacrylate photopolymerization reaction is exothermic and can locally easily reach more than 100°C.

## 5 Phase Four: Repurpose for Automation

3D printers are three‐axis robots with an extra motor to push the filament through the nozzle, and thanks to the consumer market, they are getting cheaper by the day, and it is possible to buy a 3D printer for less than €200. If you think about it, these are the most affordable three‐axis robots on the market. Just as an extreme example, if you buy a 3D printer and use it not to print but to move the printing bed back and forth with tubes, liquid cell cultures, and so on, it is way cheaper than any shaker/rocker you can buy.

The components of a 3D printer can be separated into mechanical parts and electrical components. All the 3D printers have three linear motion systems. These can be belts, rails, or lead screws, depending on the model. They have at least four stepper motors, two heating elements, two thermistors to control temperatures, three touch switches (end‐stops), plenty of screws, nuts, bolts, and all the necessary tools to assemble them. On the electronics part, they have a motherboard, a power unit, and all the cabling for connecting the motherboard to the other components. The 3D printing consumer market has grown so much that you will have a heavier bill if you attempt to buy all the single parts than buying a 3D printer.

All these goodies would be useless if we did not have a way to program and control the movements of the “robot.” This can be done by directly exploiting the 3D printer programming language. Almost all printers on the market use open‐source Marlin firmware and G‐Code as the programming language. G‐Code is highly intuitive, can be written in any text editor, and does not require any prior knowledge of programming. For example, the “G1×10 Y12 Z5” string will move the printhead to the 10/12/5 X/Y/Z space. G‐Code writing can also be automated using FullControlGCode.^[^ ^111^ ^]^

Now we have cheap mechanical and electronic components and a simple way to program and control them. This opens endless opportunities for affordable and personalized automation in the laboratory.

**Figure 5:**
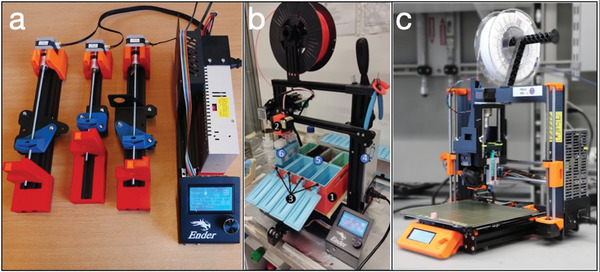
Examples of modified 3D printers. a) 3D printer mechanical and electrical components are transformed into controllable syringe pumps. Reproduced under the terms of the Creative Commons CC BY License.^[^ ^112^ ^]^ Copyright 2021, The Authors, published by Elsevier. b) A 3D printer is converted into an automatic staining machine for histology. Reproduced under the terms of the Creative Commons CC BY License.^[^ ^113^ ^]^ Copyright 2022, The Authors, published by EngRxiv. c) A modified extruder for transforming an FDM printer into a bioprinter. Reproduced under the terms of the Creative Commons CC BY License.^[^ ^114^ ^]^ Copyright 2021, The Authors, published by Elsevier.

One of the first proofs of this approach was achieved in our laboratory, where we acquired a cheap 3D printer, and with its components and some 3D printed parts, we built three programmable syringe pumps (**Figure** **5**a). These three programmable pumps cost less than half of a single, non‐programmable commercially available syringe pump.^[^ ^112^ ^]^

We also modified a 3D printer for automating histological staining procedures. Here a simple add‐on for holding glass slides is attached to the printhead. According to a histological staining procedure, the 3D printer is then programmed to move these glass slides in different solvent tanks (Figure 5b).^[^ ^113^ ^]^ This process can be easily used for programmable coating purposes as well, in contrast to high‐priced dip coating machines.

Cheap 3D printers have also been modified to print soft materials and be bioprinters at all effects (Figure 5c).^[^ ^114^ ^]^

I am convinced this is only the tip of the iceberg for automating laboratory procedures using the mechanics and electronics of 3D printers. Exploiting the consumer market for obtaining cheap and personalized laboratory equipment coupled with the expanding DIY and makers movement will result in new ideas and, at the same time, a democratization of science.

## 6 Conclusions and Outlook

To finalize, I will go through three classical questions about 3D printing in the lab. The first one is: “Which 3D printer should I buy?”

This very much depends on how you want to use the 3D printer, what is the technological level of the laboratory, and, obviously, what is the budget for it.

The Ultimaker printers are expensive but thanks to their printer/material/slicer ecosystem, they are one of the most reliable printers, even for first‐time users. They should be considered workhorses.

The Prusa's printers share a similar ecosystem of printer/material/slicer. They are more economical than the Ultimaker but still very reliable, with a great user base, good quality control, and support from the manufacturer. In United States, Flashforge printers are also labeled as reliable and with good quality control.

The Creality Ender 3 V2 is an excellent starting point for cheap printers. I recommend these printers for users who want to dig deep into the 3D printers and how they work. There is absolutely no quality control on the printers, but the user base is incredibly vast, and for each problem, you encounter, there will be plenty of solutions online. The Creality Ender 3 was one of the first 3D printers to be below €200. Nowadays, there are many clones of it, for example, from Anycubic or Voxelab.

A recommended set‐up for a lab would be to have one “indestructible” workhorse and one cheap 3D printer for learning the ins and outs of the printer and how to solve its problems.

The second question is: “What's next?”

There are a few things we can improve for making 3D printers standard equipment for laboratory use:

A proper database is still missing. So far, designs are shared on different databases, which renders the search for a particular design cumbersome. NIH made a 3D printed parts database some years ago, but many researchers prefer to use other ones. It is not well maintained, and the search for parts is almost inexistent. To solve this problem, a unified database with a good search engine and a DOI or DOI‐like system to properly cite the design is desired.

Another important thing is to teach about 3D printing and 3D design. Basic principles of 3D printing, 3D design, and programming should be in the curriculum of practically any scientist. And here, my suggestion to you is to introduce, even if for less than an hour, 3D printing in your course, regardless of what you teach. The new technology will heavily influence the future generation of scientists and introducing 3D printing and programming as soon as possible in their curricula will be of great help for students' development.

A third point to take into consideration is plastic waste. When the world is trying to reduce the use of plastic materials, 3D printing seems counter‐intuitive in terms of “green” materials. However, besides the green benefit of 3D printing in general compared to the industry standard, for example, printing only one piece, printing in the place of use, no need for shipping, and so on, green(er) materials have started appearing on the market. Recycled PETG or PLA is currently available from many different vendors, and researchers are working on upcycling 3D printable plastics.^[^ ^115^ ^]^ However, we can do better than just using recycled materials. For example, support material should be avoided as much as possible. The support material is plastic material that supports the printed parts while printing, and it will be thrown away as soon as the print finishes, so when possible, care should be taken in the design of the object to be printable without the use of any support material. A similar objection can be made for multicolor or multimaterial printers, where most of the material is purged out in “color towers.” Once again, it is waste material and should be avoided as much as possible. Plastic waste from 3D printing is only a drop in the ocean, and it is an infinitesimal part of worldwide plastic waste. However, a discussion on the topic and how to avoid unnecessary waste or minimize it is essential for an ethical research environment.

I hope I managed to convince you about the usefulness of a 3D printer in the lab. This short review focused only on FDM printers because I believe this is the first step in 3D printing and is the most versatile one. Sooner or later, you will encounter stereolithography (SLA) or masked stereolithography (m)SLA printers. They use a mixture of methacrylates and a photopolymerization reaction to manufacture a 3D object. If a student asks for these kinds of printers, buy them, they probably know more than you (and me, for what it is worth).

## Conflict of Interest

The author declares no conflict of interest.
